# Inflammation, oxidative stress, matrix metalloproteinases and DNA damage in diabetic polyneuropathy and neuropathic pain

**DOI:** 10.3389/fneur.2026.1848537

**Published:** 2026-07-20

**Authors:** Tugce Ozdemir Gultekin, Elif Gokcal, Huri Demirci, Asli Yaman Kula, Cigdem Deniz, Abdurrahim Kocyigit, Talip Asil, Azize Esra Gursoy

**Affiliations:** 1 Department of Neurology, Acibadem Maslak Hospital, Istanbul, Türkiye; 2 Department of Neurology, Faculty of Medicine, Bezmialem Vakif University, Istanbul, Türkiye; 3 Department of Neurology, Massachusetts General Hospital, Boston, MA, United States; 4 Department of Medical Biochemistry, Faculty of Medicine, Biruni University, Istanbul, Türkiye; 5 Department of Biochemistry, Faculty of Medicine, Bezmialem Vakif University, Istanbul, Türkiye

**Keywords:** diabetic distal symmetric polyneuropathy, diabetic neuropathic pain, IL-1β, IL-6, matrix metalloproteinases, oxidative stress index, TNF-α

## Abstract

**Objective:**

Diabetic polyneuropathy is one of the most common complications of type 2 diabetes mellitus (DM) and represents a major cause of morbidity and impaired quality of life. This study aimed to investigate the associations between inflammation, oxidative stress, matrix metalloproteinases (MMPs), DNA damage, and the presence of distal symmetric diabetic polyneuropathy (DPNP) and diabetic neuropathic pain (DNP).

**Methods:**

A total of 108 patients with Type 2 DM and 42 healthy controls (HC) were enrolled prospectively. Diabetic patients were classified as having DPNP based on clinical and electrodiagnostic findings. DNP was evaluated using the Douleur Neuropathique 4 (DN4) questionnaire. Serum levels of interleukin-6 (IL-6), tumor necrosis factor-*α* (TNF-*α*), interleukin-1β (IL-1β), MMP-2, MMP-9, MMP-10, Oxidative Stress Index (OSI), and DNA damage were measured.

**Results:**

Levels of IL-6, TNF-α, IL-1β, MMP-9, MMP-10, OSI, and DNA damage were significantly higher in diabetic patients compared with HC (*p* < 0.001 for all). Among diabetic patients, all biomarkers except MMP-2 were significantly elevated in those with DPNP and DNP (*p* < 0.001 for all). In diabetic patients, the levels of IL-6, TNF-α and IL-1β showed strong correlations with each other and with MMP-9 and OSI levels. Multivariable analysis demonstrated that the presence of DPNP was independently associated with increased levels of MMP-9, MMP-10, and OSI whereas the presence of DNP was independently associated with increased OSI levels.

**Conclusion:**

Inflammation, oxidative stress, and matrix metalloproteinase activation are strongly associated with DPNP and DNP in patients with Type 2 DM. Correlations among these parameters are consistent with the presence of an interconnected pathophysiological network. These findings provide additional insight into the complex pathophysiology of diabetic neuropathy.

## 1 Introduction

Diabetes mellitus (DM) is a chronic metabolic disease with a rapidly increasing global prevalence and represents a major public health concern (1). Diabetic neuropathy comprises a heterogeneous spectrum of peripheral nerve disorders, including distal symmetric polyneuropathy, entrapment neuropathies, radiculopathies, and immune-mediated forms. Distal symmetric diabetic polyneuropathy (DPNP) is the most common subtype and represents the primary driver of disability in diabetic patients (2–4). DPNP is characterized by progressive sensory and motor nerve dysfunction and is frequently accompanied by neuropathic pain, leading to substantial impairment in quality of life and increased healthcare burden (4). Recent reviews have emphasized the multifactorial nature of diabetic neuropathy, involving metabolic, inflammatory, oxidative, and neurovascular pathways (5).

The pathophysiological mechanisms underlying DPNP are complex and likely multifactorial, including inflammatory processes, oxidative stress, and mitochondrial dysfunction that causes nerve injury (6–8). Of these factors, oxidative stress is defined as a disturbance in the balance between oxidants and antioxidant capacity, resulting from either increased oxidant production or decreased antioxidant capacity (9–11).

Hyperglycemia promotes excessive generation of reactive oxygen species through several biochemical pathways, including mitochondrial dysfunction and non-enzymatic glycation of proteins. This oxidative environment may lead to structural damage in lipids, proteins, and nucleic acids. One important consequence of oxidative stress is DNA damage, which has been associated with neuronal apoptosis and impaired neuronal repair processes. The oxidative stress index (OSI), calculated as the ratio of total oxidant status (TOS) to total antioxidant status (TAS), has been proposed as a marker of oxidative stress; however, its role in diabetic neuropathy remains insufficiently explored (11).

Inflammatory processes also play an important role in the pathogenesis of diabetic neuropathy. Elevated circulating levels of pro-inflammatory cytokines such as tumor necrosis factor-*α* (TNF-α), interleukin-6 (IL-6), and interleukin-1β (IL-1β) have been reported in patients with diabetes and have been implicated in neuronal injury and peripheral nerve dysfunction. These cytokines have been proposed to be associated with nerve damage by promoting inflammatory signaling pathways, increasing oxidative stress, and impairing microvascular circulation supplying peripheral nerves (12–16).

Matrix metalloproteinases (MMPs) represent another group of molecules that have been associated with diabetic complications. MMPs are proteolytic enzymes involved in extracellular matrix remodeling and vascular structural regulation, and their dysregulation has been associated with various vascular diseases (17). Among the MMP family, MMP-2 and MMP-9 are gelatinases that have been shown to play a role in vascular remodeling and plaque stability in carotid artery atherosclerosis (18). However, studies investigating circulating levels of MMP-2 and MMP-9 in diabetic patients have reported inconsistent results (19–23). Increased levels of MMP-9 have also been described in patients with diabetic nephropathy compared with those without nephropathy (24).

MMP-10, a stromelysin involved in vascular remodeling, has been associated with microvascular complications of diabetes, including diabetic retinopathy and nephropathy (25). However, data regarding the role of MMP-10 in diabetic polyneuropathy remain limited.

Another important complication of DM is diabetic neuropathic pain (DNP), which affects approximately 20% of diabetic patients and may develop even in the absence of clinically detectable polyneuropathy (26). Although painful diabetic neuropathy is frequently associated with DPNP, not all patients with neuropathy develop pain. This observation suggests that distinct pathophysiological mechanisms may underlie neuropathy and neuropathic pain. Since most previous studies have evaluated patients with diabetic neuropathy regardless of the presence of pain, the mechanisms that differentiate painful and painless neuropathy remain insufficiently understood (27). Evaluating neuropathic pain as a distinct clinical entity may therefore provide further insights into the mechanisms of diabetic nerve injury. Recent studies have also highlighted the potential roles of small fiber dysfunction, neuroimmune signaling, and neuroinflammatory mechanisms in diabetic neuropathy. Alterations in immune–neuronal interactions and mitochondrial homeostasis have been proposed as additional contributors to peripheral nerve injury and neuropathic pain (12, 13).

Despite increasing evidence regarding the roles of oxidative stress and inflammation in diabetic neuropathy, the relationships between oxidative stress markers, DNA damage, inflammatory cytokines, and matrix metalloproteinases have not been fully clarified. In particular, limited data exist regarding the combined evaluation of these biomarkers in relation to both diabetic polyneuropathy and DNP.

Therefore, the aim of the present study was to investigate the relationship between oxidative stress, DNA damage, matrix metalloproteinases, and pro-inflammatory cytokines in patients with type 2 DM, and to evaluate their association with the presence of diabetic polyneuropathy and DNP.

## 2 Methods

### 2.1 Study design and participants

This prospective cross-sectional observational study was conducted between April 2015 and January 2016 at the Neurology Departments of a tertiary university hospital. A total of 108 patients with Type 2 DM and 42 age- and sex-matched healthy controls (HC) were included. Patients with Type 2 DM were consecutively recruited from the outpatient clinics. The diagnosis of Type 2 DM was established according to the American Diabetes Association criteria (28).

Patients aged between 18 and 80 years who were receiving antidiabetic treatment (oral agents and/or insulin) were eligible for inclusion. Patients with malignancy or genetic, autoimmune, inflammatory, or neurological diseases were excluded. In addition, participants with neuropathy attributable to causes other than diabetes (e.g., hereditary, toxic, or inflammatory), recent infection within the previous month, current or past smoking or alcohol use, or pregnancy or lactation were not included.

Healthy controls were recruited from individuals presenting for routine evaluation and were selected to achieve a comparable age and sex distribution to the diabetic cohort. No individual matching procedure was performed. Controls had no history or clinical evidence of peripheral neuropathy, and all exclusion criteria applied to the patient group were also applied to the control group.

After enrollment, demographic and clinical data were systematically recorded for all participants. All subjects underwent nerve conduction studies (NCS) as described below. NCS were performed according to standard guidelines. Written informed consent was obtained from all participants, and the study protocol was approved by the local ethics committee in accordance with the Declaration of Helsinki.

### 2.2 Assessment of diabetic polyneuropathy

All participants underwent detailed neurological examination by an experienced neurologist. Clinical data including age, sex, duration of diabetes, and current medications were recorded. Patients underwent NCS with the Keypoint Electromyography Device (Medtronic, Skovlunde, Denmark) in the Neurology Clinic Neurophysiology Laboratory. NCS were conducted with skin temperatures maintained between 31 °C and 33 °C. Motor and sensory nerve conduction studies were performed using standard techniques. Motor studies used a filter range of 10 Hz–5 kHz, a sweep speed of 5 ms/div, and a stimulus duration of 0.2 ms, whereas sensory studies used a filter range of 20 Hz to 2 kHz, a sweep speed of 1 ms/div, and a stimulus duration of 0.1 ms. The protocol for NCSs included at least unilateral studies of sural, ulnar, and median sensory nerves, as well as the peroneal, tibial, median, and ulnar motor nerves with F waves.

The diagnosis of DPNP was based on the combination of neuropathic symptoms, signs, and electrodiagnostic findings, which is recommended as the most accurate approach for clinical research purposes (29). Briefly, the presence of two or more abnormal nerve conduction results (excluding cases of median, ulnar, and peroneal nerve entrapment neuropathies), with at least one abnormality in the sural nerve, constituted the minimum diagnostic criteria for electrophysiological studies. Nerve conduction abnormalities were interpreted according to age-adjusted laboratory normative values and the electrodiagnostic criteria described by England et al. The diagnostic approach was also consistent with the recommendations of the Toronto Diabetic Neuropathy Expert Group (29, 30).

Patients were classified as DPNP positive ‘[DPNP(+)] if clinical findings of peripheral neuropathy were accompanied by abnormal electrophysiological findings consistent with polyneuropathy. Patients without clinical or electrophysiological evidence of neuropathy were classified as DPNP negative [DPNP(−)].

### 2.3 Assessment of diabetic neuropathic pain

Neuropathic pain was evaluated using the Douleur Neuropathique 4 (DN4) questionnaire, a validated screening tool with reported sensitivity of 80%, specificity of 92%, and a positive predictive value of 82% at a cut-off score of 4 (31). Patients were classified as having DNP if the DN4 score was ≥4 and as not having DNP if the DN4 score was <4.

### 2.4 Assessment of DNA damage

DNA damage was assessed using the alkaline single-cell gel electrophoresis (Comet) assay. Peripheral blood lymphocytes were isolated, embedded in low-melting-point agarose on microscope slides, and subjected to alkaline lysis at 4 °C for 50 min. Following lysis, slides were incubated in alkaline electrophoresis buffer for 40 min and electrophoresed at 25 V and 300 mA for 18 min. The slides were subsequently stained with ethidium bromide (2 mg/mL) and examined under a fluorescence microscope (Leica DM1000 LED, Germany). DNA damage was quantified using Comet Assay IV image analysis software (Perceptive Instruments, United Kingdom). The percentage of DNA in the comet tail (% Tail DNA) was calculated for each cell and used as the primary indicator of DNA damage. Higher % Tail DNA values were interpreted as reflecting greater levels of DNA strand breaks (32). All DNA damage analyses were performed by investigators blinded to the clinical status of the participants.

### 2.5 Biochemical analysis

Peripheral venous blood samples were obtained from all participants in the morning after an overnight fasting period of at least 12 h. Levels of glycated hemoglobin (HbA1c), low-density lipoprotein (LDL), high-density lipoprotein (HDL), triglycerides (TG), and total cholesterol were measured. Serum concentrations of interleukin-1β (IL-1β), interleukin-6 (IL-6), tumor necrosis factor-*α* (TNF-α), matrix metalloproteinase-2 (MMP-2), MMP-9, and MMP-10 were determined using enzyme-linked immunosorbent assay (ELISA). Serum concentrations of IL-1β (eBioscience, Cat. No. BMS224/2), IL-6 (eBioscience, Cat. No. BMS213/2), TNF-α (eBioscience, Cat. No. BMS223/4), MMP-2 (SunRed Biotechnology, Cat. No. 201–12-0905), MMP-9 (eBioscience, Cat. No. BMS2016/2), and MMP-10 (SunRed Biotechnology, Cat. No. 201–12-0906) were determined using commercially available ELISA kits and analyzed with a Thermo Scientific Multiskan FC microplate reader (United States). According to the manufacturers’ specifications, the intra-assay and inter-assay coefficients of variation were <10 and <12%, respectively. Measurements were performed according to the manufacturers’ instructions using calibration curves generated from serial dilutions of the supplied standards.

Total antioxidant status (TAS) and total oxidant status (TOS) were measured using automated colorimetric methods at wavelengths of 240 nm and 520 nm, respectively (33, 34). The oxidative stress index (OSI) was calculated as the ratio of TOS to TAS according to the following formula: OSI = (TOS [μmol H₂O₂ equivalent/L] × 100) / TAS [μmol Trolox equivalent/L] (35).

### 2.6 Statistical analysis

Normality of continuous variables was assessed using the Shapiro–Wilk test together with visual inspection of histograms and Q–Q plots. Continuous variables were expressed as median (minimum–maximum) or median (interquartile range), depending on their distribution. Group comparisons were performed using the independent-samples *t*-test or Mann–Whitney *U* test for two groups and the Kruskal–Wallis test for multiple groups. Categorical variables were analyzed using Fisher’s exact test. To account for multiple testing, Bonferroni correction was applied to the primary biomarker comparisons. For the primary biomarker comparisons, the Bonferroni-corrected significance threshold was *p* < 0.005.

Correlation analyses were performed using Spearman’s correlation coefficient. Multivariable logistic regression models were constructed to identify factors independently associated with DPNP and DNP. Variables with a *p*-value <0.2 in univariate analyses, as well as age, were included in the models. Pro-inflammatory cytokines and DNA damage were excluded due to significant multicollinearity.

Statistical analyses were performed using SPSS version 23. A two-tailed *p*-value <0.05 was considered statistically significant.

## 3 Results

### 3.1 Study population

The study cohort comprised 150 participants: 108 with Type 2 DM and 42 HC. The mean age of the overall cohort was 55 years, and 61.3% were female. Age and sex distribution did not differ significantly between diabetic patients and controls (*p* = 0.230 and *p* = 0.226, respectively).

As expected, HbA1c levels were significantly higher in patients with diabetes compared with controls (*p* < 0.001). Triglyceride levels were also higher, while HDL levels were lower in diabetic patients (p < 0.001 and *p* = 0.004, respectively). LDL levels were higher in patients; however, this difference did not reach statistical significance (*p* = 0.078).

HbA1c levels showed a stepwise increase from controls to DPNP(−) and DPNP(+) groups. Triglyceride levels also showed a stepwise increase across groups, whereas HDL levels demonstrated a decreasing trend. Clinical and laboratory characteristics of the study groups are presented in Table 1.

**Table 1:** Clinical and laboratory characteristics of the study groups.

Variable	Control, *n* = 42 Median (min–max)	DPNP(−), *n* = 56 Median (min–max)	DPNP(+), *n* = 52 Median (min–max)	*p*
Age (years), median (min–max)	52 (40–70)	55 (34–75)	58 (40–70)	0.154
Female, *n* (%)	29 (69%)	35 (63%)	28 (54%)	0.31
Hypertension, *n* (%)	–	24 (43%)	27 (52%)	0.35
Duration of diabetes (years), median (min–max)	–	6 (1–30)	15 (1–40)	<0.001
HbA1c (%)	5.54 (4.81–6.42)	6.75 (5.5–11.5)	8.28 (5.37–12.06)	<0.001
HDL (mg/dL)	49.5 (34–70.3)	45.95 (26–96)	42.8 (28–68)	0.002
LDL (mg/dL)	109 (55–167)	112.5 (71–253)	117 (54–215)	0.198
TG (mg/dL)	112.5 (60–276)	149 (60–564)	178.5 (73–517)	0.000
Total cholesterol (mg/dL)	195 (127–268)	201 (135–372)	198 (106–320)	0.337

Data are presented as median (min–max) or *n* (%). *p*-values were calculated using the Kruskal–Wallis test or chi-square test, as appropriate.

The mean duration of diabetes was 11.4 years. Regarding treatment, 56 patients were receiving oral antidiabetic agents, 8 were treated with insulin only, and 44 were receiving a combination of oral agents and insulin.

Hypertension was present in 47.2% of diabetic patients. Duration of diabetes, HbA1c levels, and lipid parameters did not differ between patients with and without hypertension (*p* > 0.2 for all).

### 3.2 Diabetic polyneuropathy and neuropathic pain

Distal symmetric diabetic polyneuropathy (DPNP) was present in 52 patients [DPNP(+)], while 56 patients had no evidence of DPNP [DPNP(−)]. Patients with DPNP had a significantly longer duration of diabetes compared with those without DPNP (*p* < 0.001). Age was slightly higher in the DPNP(+) group; however, this difference did not reach statistical significance (*p* = 0.094). Sex distribution was similar between the groups (*p* = 0.362).

Clinical and biomarker characteristics according to the presence of DPNP and DNP are summarized in Tables 2, 3.

**Table 2:** The comparison of biomarker levels across study groups.

Variable	Control, *n* = 42 Median (min–max)	DPNP(−), *n* = 56 Median (min–max)	DPNP(+), *n* = 52 Median (min–max)	*p*
Vit D (ng/mL)	10.48 (2.69–58.28)	8.62 (1.16–57.53)	7.93 (1.78–39.78)	0.143
IL-6 (pg/mL)	18.11 (9.32–30.15)	54.10 (21.10–65.32)	75.11 (58.11–93.10)	<0.001
TNF-α (pg/mL)	90.57 (46.58–150.75)	270.51 (105.50–326.58)	375.53 (290.55–465.49)	<0.001
IL-1β (pg/mL)	40.46 (2.87–64.54)	112.44 (46.44–134.87)	154.46 (120.46–190.43)	<0.001
MMP-2 (ng/mL)	42.73 (28.70–1530.15)	745.40 (23.55–8956.23)	699.17 (35.81–1831.56)	<0.001
MMP-9 (ng/mL)	3.14 (1.02–6.57)	7.77 (5.21–12.87)	12.57 (9.02–19.46)	<0.001
MMP-10 (ng/mL)	6.00 (2.94–9.76)	16.51 (10.90–31.50)	32.65 (14.80–44.65)	<0.001
TAS (μmol Trolox equiv./L)	1.84 (0.79–2.09)	0.93 (0.67–1.95)	0.84 (0.62–1.03)	<0.001
TOS (μmol H_2_O_2_ equiv./L)	7.89 (6.17–9.94)	9.60 (7.19–12.85)	11.33 (7.69–14.10)	<0.001
OSI	4.67 (3.33–11.11)	10.41 (5.54–14.40)	13.36 (9.46–20.00)	<0.001
DNA Damage	24.70 (14.33–31.29)	41.08 (30.66–63.78)	59.23 (31.51–71.89)	<0.001

**Table 3:** Comparison of biomarker levels according to the presence of diabetic neuropathic pain.

Variable	Patients with neuropathic pain (*n* = 48)	Patients without neuropathic pain (*n* = 60)	*P*
IL-6 (pg/mL)	72.2 (63–78)	58.1 (46–68)	<0.001
TNF-α (pg/mL)	358.6 (316–388)	290.6 (230–339)	<0.001
IL-1β (pg/mL)	148.6 (130–160)	120.5 (96–139)	<0.001
MMP-9 (ng/mL)	11.5 (10–13)	8.5 (7–10)	<0.001
MMP-10 (ng/mL)	31.6 (26–34)	19.7 (15–29)	<0.001
MMP-2 (ng/mL)	767 (459–1,121)	729 (455–920)	0.357
OSI	12.8 (12–14)	10.4 (9–12)	<0.001

DNP, diabetic neuropathic pain; MMP, matrix metalloproteinase; OSI, oxidative stress index; IL, interleukin. Data are presented as median (IQR, interquartile range). *P*-values were calculated using the Mann–Whitney *U* test.

HbA1c levels were significantly higher in patients with DPNP compared with those without DPNP (*p* < 0.001). HDL levels were lower in patients with DPNP; however, this difference did not reach statistical significance between DPNP(+) and DPNP(−) patients. There were no significant differences in triglyceride or LDL levels between the groups.

Biomarker analysis showed that IL-6, TNF-*α*, IL-1β, MMP-9, MMP-10, TOS, and OSI levels were significantly higher in patients with DPNP compared with those without DPNP (*p* < 0.001 for all), whereas TAS levels were significantly lower (*p* < 0.001). MMP-2 levels did not differ between the groups. DNA damage levels were also significantly higher in patients with DPNP (*p* < 0.001).

A total of 48 patients (44.4%) were classified as having diabetic neuropathic pain (DNP+). Among these patients, 71% were in the DPNP(+) group, while 20% were in the DPNP(−) group.

Pro-inflammatory cytokines (IL-6, TNF-α, IL-1β), MMP-9, MMP-10, TOS, and OSI levels were significantly higher in patients with DNP (*p* < 0.001 for all), whereas TAS levels were significantly lower (*p* = 0.002). DNA damage levels were significantly higher in patients with DNP (*p* < 0.001).

No significant differences were observed between DNP+ and DNP– groups in terms of HDL, LDL, triglycerides, total cholesterol, vitamin D, or MMP-2 levels (*p* > 0.05 for all comparisons).

### 3.3 The comparison of biomarkers between patients and healthy controls

Compared with healthy controls, diabetic patients had significantly higher levels of pro-inflammatory cytokines (IL-6, TNF-α, IL-1β), MMP-2, MMP-9, MMP-10, TOS, OSI, and DNA damage (*p* < 0.001 for all), whereas TAS levels were significantly lower. A stepwise increase in inflammatory, oxidative, and matrix-related biomarkers was observed from healthy controls to DPNP(−) and DPNP(+) patients.

**Figure 1:**
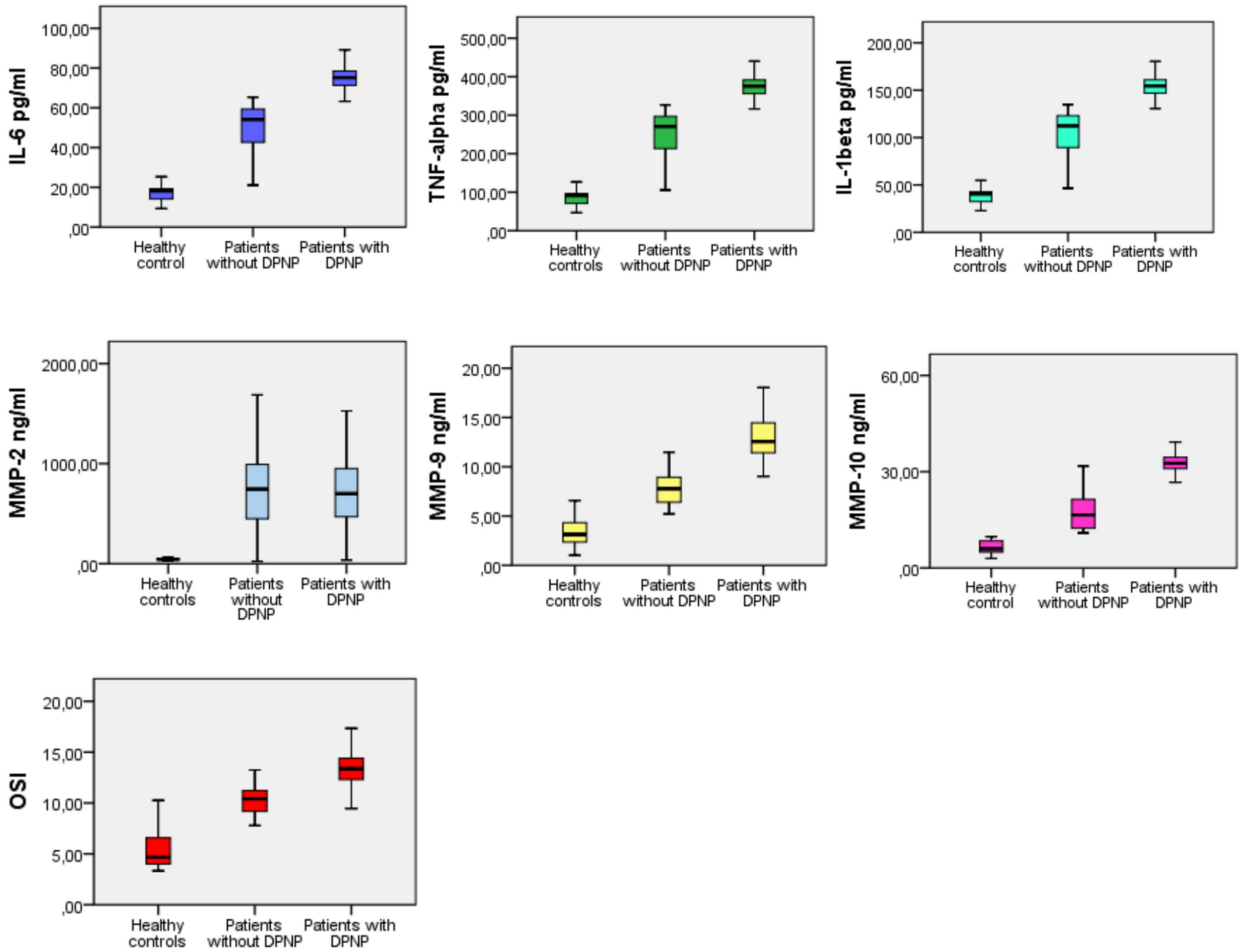
Comparison of biomarker levels across healthy controls (HC), diabetic patients without distal symmetric diabetic polyneuropathy [DPNP(−)], and diabetic patients with distal symmetric diabetic polyneuropathy [DPNP(+)]. IL-6, interleukin-6; TNF-α, tumor necrosis factor-α; IL-1β, interleukin-1β; MMP-2, matrix metalloproteinase-2; MMP-9, matrix metalloproteinase-9; MMP-10, matrix metalloproteinase-10; OSI, oxidative stress index. Bars represent median values and error bars indicate interquartile ranges (IQR). Overall group comparisons were performed using the Kruskal–Wallis test. All comparisons shown were statistically significant (*p* < 0.001). Detailed numerical results are presented in Table 2.

As shown in Figure 1, these biomarkers demonstrated a stepwise increase from healthy controls to DPNP(−) patients and further to DPNP(+) patients, indicating a progressive association with neuropathy. All significant biomarker differences remained statistically significant after Bonferroni correction for multiple comparisons.

Patients with neuropathic pain had significantly higher levels of inflammatory cytokines, MMP-9, MMP-10, and OSI compared with those without neuropathic pain.

Correlation analyses of biomarkers are presented in the Supplementary material.

### 3.4 Multivariable analysis

Multivariable logistic regression results are presented in Table 4. In multivariable logistic regression analysis adjusted for age, duration of diabetes, HbA1c, and HDL levels, DPNP was independently associated with increased levels of MMP-9 (OR 5.8, 95% CI 1.7–19.4, *p* = 0.004), OSI (OR 4.3, 95% CI 1.1–16.6, *p* = 0.033), and MMP-10 (OR 1.1, 95% CI 1.0–1.1, *p* = 0.034).

**Table 4:** Multivariable logistic regression analysis of factors independently associated with diabetic neuropathic pain (DNP).

Variable	Adjusted Odds Ratio (95% CI)	*p*
MMP-9	1.1 (0.9–1.3)	0.143
MMP-10	1.1 (0.9–1.1)	0.922
OSI	1.4 (1.1–1.8)	0.038

MMP, matrix metalloproteinase; OSI, oxidative stress index.

Adjusted for age, duration of diabetes, and the presence of diabetic polyneuropathy (DPNP).

In a separate model adjusted for age, duration of diabetes, and DPNP, OSI levels were independently associated with DNP (OR 1.4, 95% CI 1.1–1.8, *p* = 0.038).

In patients without DPNP, OSI levels remained significantly higher in those with neuropathic pain (*p* = 0.013), and OSI was independently associated with DNP in this subgroup (OR 1.7, 95% CI 1–3, *p* = 0.035).

## 4 Discussion

This study demonstrates that circulating inflammatory cytokines, matrix metalloproteinases (MMPs), oxidative stress markers, and DNA damage are elevated in diabetic patients compared with controls. Among diabetic individuals, all biomarkers except MMP-2 were higher in those with DPNP and DNP. Multivariable analyses identified MMP-9, MMP-10, and OSI as independent correlates of DPNP, while only OSI was independently linked to DNP. Notably, oxidative stress was associated with neuropathic pain even in the absence of clinically detectable polyneuropathy. To our knowledge, this is one of the few studies simultaneously evaluating DNA damage, MMP-10, and inflammatory and oxidative markers in relation to both DPNP and neuropathic pain.

Inflammation has been consistently associated with diabetic neuropathy and is considered one of the potential mechanisms involved in its pathophysiology. Consistent with previous studies, we observed significantly higher levels of IL-6, TNF-α, and IL-1β in diabetic patients, with further increases in patients with DPNP and DNP. These cytokines have been implicated in neuronal injury through inflammatory signaling pathways, increased oxidative stress, and microvascular dysfunction (15, 16, 36–42). Importantly, our findings extend the existing literature by demonstrating a progressive increase in cytokine levels from diabetic patients without neuropathy to those with neuropathy and neuropathic pain, suggesting that inflammation may be associated not only with nerve injury but also with neuropathic pain.

Matrix metalloproteinases represent another important component of the pathological cascade. In our study, MMP-2, MMP-9, and MMP-10 levels were elevated in diabetic patients compared with healthy controls, consistent with previous studies reporting increased circulating MMP levels in diabetes. However, only MMP-9 and MMP-10 were associated with DPNP and DNP, whereas MMP-2 did not differ between neuropathy subgroups. These findings suggest that MMP-2 reflects systemic metabolic and vascular alterations, whereas MMP-9 and MMP-10 showed stronger associations with DPNP and DNP than MMP-2 in the present study. While prior studies have primarily focused on MMP-9 in diabetic complications, data regarding MMP-10 are limited (23, 43–46). It should be noted that previous studies investigating circulating MMP levels in diabetes have reported inconsistent findings. While several studies demonstrated increased MMP-2 and MMP-9 levels in diabetic patients, others failed to identify significant associations with diabetic complications or vascular parameters (19–23). Differences in study populations, metabolic characteristics, and study methodologies may partly explain the heterogeneity of reported findings (47). These observations suggest that different MMP family members may reflect distinct biological processes in diabetes and its complications (47, 48). The lack of association between MMP-2 and DPNP may reflect biological differences among MMP family members. MMP-2 is constitutively expressed in many tissues and has been associated with systemic vascular and metabolic alterations in diabetes. In contrast, MMP-9 and MMP-10 are more closely linked to inflammatory activation and tissue remodeling. To our knowledge, this is among the first studies to evaluate circulating MMP-10 levels in relation to diabetic polyneuropathy. Although the underlying mechanisms cannot be determined from the present data, the observed association extends the current literature and supports further investigation of MMP-10 in diabetic neuropathy. Experimental studies have suggested that MMPs may influence extracellular matrix remodeling, vascular permeability, and inflammatory cell migration. Through these mechanisms, altered MMP activity has been proposed to affect the microenvironment surrounding peripheral nerves and the integrity of the blood–nerve barrier, potentially facilitating neuroinflammatory processes (48). Inflammatory cytokines and matrix metalloproteinases are closely interconnected biological systems. Pro-inflammatory cytokines have been shown to regulate MMP expression, whereas MMP activation may further amplify inflammatory signaling through extracellular matrix remodeling and cytokine processing (47, 49).

Oxidative stress showed the strongest and most consistent association with both DPNP and DNP in our study. OSI levels were significantly higher in diabetic patients, further increased in those with DPNP and DNP, and independently associated with both conditions. These findings are in agreement with previous studies demonstrating increased oxidative stress in diabetic neuropathy (50–58). Importantly, our results further indicate that oxidative stress is independently associated with neuropathic pain, even in the absence of clinically detectable neuropathy.

In addition to inflammatory and oxidative mechanisms, metabolic factors have also been associated with diabetic neuropathy. In our study, HbA1c levels were significantly higher in patients with DPNP than in DPNP(−) patients and healthy controls, supporting an association between poor glycemic control and diabetic neuropathy (30, 59, 60). This finding is consistent with previous studies demonstrating an association between chronic hyperglycemia and the development and severity of diabetic neuropathy. HDL levels were lower in patients with DPNP compared with controls, whereas triglyceride levels were higher in diabetic patients overall but did not differ between these groups. Taken together, these findings suggest that while dyslipidemia is a feature of diabetes, its association with diabetic polyneuropathy appears to be less specific than that of hyperglycemia. In contrast, inflammatory and oxidative stress markers showed stronger associations with DPNP in the present study.

Another important finding of this study is the increase in DNA damage in patients with DPNP and DNP. Chronic hyperglycemia-induced oxidative stress has been associated with DNA strand breaks and impaired cellular repair, contributing to neuronal dysfunction. Although previous studies have linked oxidative stress to neuronal injury, data on DNA damage in diabetic neuropathy remain limited (61–64). In this context, our findings provide additional evidence supporting DNA damage as a downstream marker of oxidative stress–mediated neuronal injury. Although DNA damage was not included in multivariable models due to its close association with oxidative stress parameters, its consistent elevation across patient groups suggests its potential relevance to the pathophysiology of diabetic neuropathy. However, the present study did not evaluate DNA repair pathways, mitochondrial function, or apoptosis-related mechanisms.

The differential findings between DPNP and DNP are particularly noteworthy. While both conditions were associated with increased inflammation, oxidative stress, and MMP activation, only oxidative stress remained independently associated with neuropathic pain. These findings suggest that neuropathy and neuropathic pain, although related, may involve partially distinct biological mechanisms, with oxidative stress showing the strongest independent association with neuropathic pain. This distinction is supported by previous studies indicating partially distinct mechanisms between painful and painless neuropathy and may have important implications for the development of targeted therapeutic strategies (26).

This study has several strengths. First, it simultaneously evaluated multiple biological pathways—including inflammation, oxidative stress, matrix remodeling, and DNA damage—in a well-characterized cohort. Second, it assessed diabetic polyneuropathy and neuropathic pain as distinct clinical entities, allowing for a clearer differentiation between these related conditions. Third, the use of multivariable analyses enabled identification of biomarkers independently associated with clinical outcomes.

However, several limitations should be considered. This was a prospective cross-sectional observational study; therefore, causal relationships cannot be established. The sample size, particularly in subgroup analyses, was relatively limited. In addition, no formal *a priori* sample size calculation was performed because the study was originally designed as an exploratory biomarker study; therefore, some subgroup analyses may have been underpowered. Certain potential confounders, such as diabetic nephropathy and detailed metabolic parameters, were not evaluated. Factors such as body mass index, renal function, metabolic syndrome, dietary habits, physical activity, and socioeconomic status were not systematically assessed. These factors may influence inflammatory and oxidative stress markers and therefore could have affected the observed associations. DNA damage was not included in regression analyses, which may limit conclusions regarding its independent contribution. The potential modifying effects of antihyperglycemic and other concomitant medications were not specifically evaluated. Although treatment modality (oral antidiabetic agents, insulin, or combination therapy) was recorded, medication-specific analyses were not performed; therefore, the potential impact of treatment differences on biomarker levels cannot be excluded. Patients with isolated small fiber neuropathy may have been misclassified as DPNP(−), as the diagnosis of DPNP relied primarily on nerve conduction studies. Because small fiber dysfunction may precede electrophysiological abnormalities, the observed biomarker associations may have been underestimated. Quantitative sensory testing, skin biopsy, and corneal confocal microscopy were not performed. In addition, the present study was not designed to evaluate diagnostic thresholds, predictive performance, or longitudinal clinical utility of the investigated biomarkers.

In conclusion, inflammation, oxidative stress, matrix metalloproteinase activation, and DNA damage are closely associated with diabetic polyneuropathy and neuropathic pain. Among these mechanisms, oxidative stress showed the strongest association with both diabetic polyneuropathy and neuropathic pain, particularly with neuropathic pain. These findings further highlight the complex interplay between inflammatory, oxidative, and matrix-remodeling pathways in diabetic neuropathy.

## Data Availability

The raw data supporting the conclusions of this article will be made available by the authors, without undue reservation.
