# Supplementary material for: Inflammation, oxidative stress, matrix metalloproteinases and DNA damage in diabetic polyneuropathy and neuropathic pain
Source: Front Neurol. 2026 Jul 20;17:1848537. doi: 10.3389/fneur.2026.1848537 (PMC13432961; doi:10.3389/fneur.2026.1848537)
Supplement: Supplementary file 1 [file Supplementary_file_1.DOCX]

**Supplementary Material**

**Supplementary Table 1.** Correlation of biomarker levels with each other in the whole cohort, corrected for age

|  | Control  Variables | HbA1c | HDL | LDL | TG | IL-6 | TNF-α | IL-1 β | MMP-9 | MMP-2 | MMP-10 | OSI |
| --- | --- | --- | --- | --- | --- | --- | --- | --- | --- | --- | --- | --- |
| HbA1c | (r) | 1 | -0.218 | 0.092 | 0.199 | 0.592 | 0.596 | 0.593 | 0.588 | 0.188 | 0.405 | 0.592 |
|  | p |  | 0.008 | 0.266 | 0.015 | <0.001 | <0.001 | <0.001 | <0.001 | 0.022 | <0.001 | <0.001 |
| HDL | (r) | -0.218 | 1 | 0.017 | -0.354 | -0.249 | -0.248 | -0.249 | -0.25 | -0.052 | -0.095 | -0.179 |
|  | p | 0.008 | . | 0.833 | <0.001 | 0.002 | 0.002 | 0.002 | 0.002 | 0.531 | 0.248 | 0.029 |
| LDL | (r) | 0.092 | 0.017 | 1 | 0.276 | 0.123 | 0.122 | 0.123 | 0.135 | 0.186 | 0.066 | 0.144 |
|  | p | 0.266 | 0.833 | . | 0.001 | 0.135 | 0.138 | 0.136 | 0.1 | 0.023 | 0.427 | 0.079 |
| TG | (r) | 0.199 | -0.354 | 0.276 | 1 | 0.285 | 0.287 | 0.286 | 0.248 | 0.137 | 0.142 | 0.211 |
|  | p | 0.015 | <0.001 | 0.001 | . | <0.001 | <0.001 | <0.001 | 0.002 | 0.096 | 0.084 | 0.01 |
| IL-6 | (r) | 0.592 | -0.249 | 0.123 | 0.285 | 1 | 0.999 | 1 | 0.829 | 0.28 | 0.319 | 0.83 |
|  | p | 0 | 0.002 | 0.135 | <0.001 | . | <0.001 | <0.001 | <0.001 | 0.001 | 0 | 0 |
| TNF-α | (r) | 0.596 | -0.248 | 0.122 | 0.287 | 0.999 | 1 | 1 | 0.829 | 0.281 | 0.318 | 0.831 |
|  | p | 0 | 0.002 | 0.138 | <0.001 | <0.001 | . | <0.001 | <0.001 | 0.001 | <0.001 | <0.001 |
| IL-1 β | (r) | 0.593 | -0.249 | 0.123 | 0.286 | 1 | 1 | 1 | 0.829 | 0.28 | 0.319 | 0.83 |
|  | p | <0.001 | 0.002 | 0.136 | <0.001 | <0.001 | <0.001 | . | <0.001 | 0.001 | <0.001 | <0.001 |
| MMP-9 | (r) | 0.588 | -0.25 | 0.135 | 0.248 | 0.829 | 0.829 | 0.829 | 1 | 0.232 | 0.327 | 0.794 |
|  | p | <0.001 | 0.002 | 0.100 | 0.002 | <0.001 | <0.001 | <0.001 | . | 0.004 | <0.001 | <0.001 |
| MMP-2 | (r) | 0.188 | -0.052 | 0.186 | 0.137 | 0.28 | 0.281 | 0.28 | 0.232 | 1 | 0.143 | 0.274 |
|  | p | 0.022 | 0.531 | 0.023 | 0.096 | 0.001 | 0.001 | 0.001 | 0.004 | . | 0.082 | 0.001 |
| MMP-10 | (r) | 0.405 | -0.095 | 0.066 | 0.142 | 0.319 | 0.318 | 0.319 | 0.327 | 0.143 | 1 | 0.313 |
|  | p | <0.001 | 0.248 | 0.427 | 0.084 | <0.001 | <0.001 | <0.001 | <0.001 | 0.082 | . | <0.001 |
| OSI | (r) | 0.592 | -0.179 | 0.144 | 0.211 | 0.83 | 0.831 | 0.83 | 0.794 | 0.274 | 0.313 | 1 |
|  | p | <0.001 | 0.029 | 0.079 | 0.01 | <0.001 | <0.001 | <0.001 | <0.001 | 0.001 | <0.001 | . |

Spearman correlation coefficients, (r); Hemoglobin A1c, HbA1c; high-density lipoprotein, HDL; interleukin, IL; low-density lipoprotein, LDL; matrix metalloproteinase, MMP; oxidative stress index, OSI; total antioxidant status, TAS; total oxidant status, TOS; triglycerides, TG; tumor necrosis factor-α (TNF-α).

**Supplementary Table 2.** Correlation of biomarker levels with each other in diabetic patients, corrected for age and duration of diabetes

|  | Control  Variables | HbA1c | HDL | LDL | TG | IL-6 | TNF-α | IL-1 β | MMP-9 | MMP-2 | MMP-10 | OSI |
| --- | --- | --- | --- | --- | --- | --- | --- | --- | --- | --- | --- | --- |
| HbA1c | (r) | 1 | -0.143 | -0.02 | 0.035 | 0.153 | 0.165 | 0.157 | 0.203 | 0.258 | -0.061 | 0.221 |
|  | p | . | 0.143 | 0.84 | 0.724 | 0.118 | 0.091 | 0.109 | 0.037 | 0.008 | 0.537 | 0.023 |
| HDL | (r) | -0.143 | 1 | 0.047 | -0.345 | -0.161 | -0.16 | -0.161 | -0.218 | -0.027 | 0.013 | -0.024 |
|  | p | 0.143 | . | 0.634 | <0.001 | 0.099 | 0.102 | 0.1 | 0.025 | 0.782 | 0.894 | 0.809 |
| LDL | (r) | -0.02 | 0.047 | 1 | 0.209 | -0.035 | -0.037 | -0.036 | 0.01 | 0.001 | 0.138 | 0.022 |
|  | p | 0.84 | 0.634 | . | 0.031 | 0.721 | 0.709 | 0.717 | 0.916 | 0.992 | 0.157 | 0.823 |
| TG | (r) | 0.035 | -0.345 | 0.209 | 1 | 0.093 | 0.099 | 0.095 | 0.044 | 0.036 | 0.017 | -0.067 |
|  | p | 0.724 | <0.001 | 0.031 | . | 0.343 | 0.312 | 0.333 | 0.656 | 0.714 | 0.859 | 0.494 |
| IL-6 | (r) | 0.153 | -0.161 | -0.035 | 0.093 | 1 | 0.998 | 1 | 0.559 | 0.026 | -0.123 | 0.539 |
|  | p | 0.118 | 0.099 | 0.721 | 0.343 | . | <0.001 | <0.001 | <0.001 | 0.791 | 0.208 | <0.001 |
| TNF-α | (r) | 0.165 | -0.16 | -0.037 | 0.099 | 0.998 | 1 | 0.999 | 0.562 | 0.024 | -0.121 | 0.544 |
|  | p | 0.091 | 0.102 | 0.709 | 0.312 | <0.001 | . | <0.001 | <0.001 | 0.806 | 0.217 | <0.001 |
| IL-1 β | (r) | 0.157 | -0.161 | -0.036 | 0.095 | 1 | 0.999 | 1 | 0.56 | 0.025 | -0.123 | 0.541 |
|  | p | 0.109 | 0.100 | 0.717 | 0.333 | <0.001 | <0.001 | . | <0.001 | 0.796 | 0.211 | <0.001 |
| MMP-9 | (r) | 0.203 | -0.218 | 0.010 | 0.044 | 0.559 | 0.562 | 0.56 | 1 | 0.09 | -0.109 | 0.552 |
|  | p | 0.037 | 0.025 | 0.916 | 0.656 | <0.001 | <0.001 | <0.001 | . | 0.36 | 0.268 | <0.001 |
| MMP-2 | (r) | 0.258 | -0.027 | 0.001 | 0.036 | 0.026 | 0.024 | 0.025 | 0.09 | 1 | -0.001 | 0.056 |
|  | p | 0.008 | 0.782 | 0.992 | 0.714 | 0.791 | 0.806 | 0.796 | 0.36 | . | 0.995 | 0.565 |
| MMP-10 | (r) | -0.061 | 0.013 | 0.138 | 0.017 | -0.123 | -0.121 | -0.123 | -0.109 | -0.001 | 1 | -0.077 |
|  | p | 0.537 | 0.894 | 0.157 | 0.859 | 0.208 | 0.217 | 0.211 | 0.268 | 0.995 | . | 0.433 |

Spearman correlation coefficients, (r); Hemoglobin A1c, HbA1c; high-density lipoprotein, HDL; interleukin, IL; low-density lipoprotein, LDL; matrix metalloproteinase, MMP; oxidative stress index, OSI; total antioxidant status, TAS; total oxidant status, TOS; triglycerides, TG; tumor necrosis factor-α (TNF-α).
